# New ultrasound-assisted microreactor for extracting extraterrestrial biomolecules

**DOI:** 10.1016/j.ultsonch.2025.107416

**Published:** 2025-06-03

**Authors:** Ramzi Timoumi, Rihab Fkiri, Prince Amaniampong, Guillaume Rioland, Brian Gregoire, Pauline Poinot, Claude Geffroy-Rodier

**Affiliations:** a UMR CNRS 7285, Institut de Chimie des Milieux et Matériaux de Poitiers (IC2MP), University of Poitiers, 4 rue Michel-Brunet, TSA 51106, 86073 Poitiers Cedex 9, France; b Centre National d’Etudes Spatiales (CNES), Service Laboratoires & Expertise, 18 Avenue Edouard Belin, CEDEX 9, 61401 Toulouse, France

**Keywords:** Microreactor, One-step ultrasound-assisted extraction and derivatisation, Amino acid, Meteorite

## Abstract

Searching for organics in extraterrestrial environments, especially biomolecules, continues to be a notable challenge for *in situ* missions. To address this challenge, this paper introduces an ultrasound (US)-assisted reactor for extraction of amino acids, which complies with space constraints.. The optimal duration for *in situ* extraction was set at 10 min, using a mixture of water and methanol (1:1, v/v). Compared to 24 h hot water extraction, a 10-min US extraction at 2.4 MHz or 20 kHz enable 80 % of the amino acid recovery from the Mukundpura meteorite and 100 % recovery was achieved when US-assisted extraction was performed three times. Subsequently, the 10-min US extraction at 2.4 MHz was performed in the presence of methyl chloroformate, a derivatisation agent necessary for the gas chromatography–mass spectrometry analysis of amino acids. This simultaneous extraction-derivatisation method, lasting 10 min, enabled the recovery of 80 % of the amino acid content relative to that attainable using the extraction followed by derivatisation method. This simple, rapid, and efficient method enables for the first time the the extraction and detection of amino acids from rock samples. Detection of such biomolecules on planetary or comets surfaces will be a major advance in the understanding of the origins of the first building blocks.

## 1 Introduction

In recent years, the exploration of biomolecules in mineral samples has garnered increasing attention owing to both industrial and environmental considerations and for conducting astrobiological research. *In situ* analysis of planets or comets has become essential for swiftly gathering results to guide the selection of relevant samples for future laboratory analyses. Although *in situ* analyses have enabled the detection of organics, nearly none of them have been confirmed as biomolecules [[1], [2], [3]]. In contrast, a diverse and complex organic composition, with numerous amino acids and nucleobases, has been detected in carbonaceous meteorites and their parent bodies [[4], [5], [6], [7], [8], [9], [10]]. To date, glycine represents the sole biomolecule detected *in situ,* having been found in the interstellar medium and within the icy body of 67P/Churyumov-Gerasimenko coma, though never in planetary samples [11]. The absence of glycine in planet samples could be attributed to the lack of an extraction step in space missions, despite its importance in enhancing the detectability of molecules [12,13]. Considering this aspect, the parameters of a focused ultrasound-assisted extraction method were optimised in this work [14]. Although focused ultrasonic probes have been considered for *in situ* analysis, they remain to be implemented [15]. Ultrasonic baths, commonly used for conducting validation tests of space instrumentation, are not suitable for automation and cannot satisfy space constraints [12,13,16]. To overcome these obstacles, we propose a piezoelectric microreactor as an alternative. This miniaturised system can deliver intense shockwaves to the solid matrix, resulting in improved fragmentation and mass transfer. Notably, we outline the specifications for a 2.4 MHz device designed to closely resemble the sample treatment pilot previously used in the Sample Analysis at Mars experiment on the Mars Sample Laboratory [13,17]. For validation, to mimic space instrumentation, analysis is performed through gas chromatography coupled with mass spectrometry (GC–MS), the only chromatographic method validated for *in situ* applications [[18], [19], [20], [21]]. For targeted biomolecules, derivatisation is needed prior to GC–MS analysis, which increases the number of steps to reach ready-to-inject compounds. The sample treatment, already implemented in *in situ* Martian instrumentation, is expected to be considerably enhanced through an extraction step [17,22,23].

Overall, this work is aimed at investigating the efficiency of the 2.4 MHz extraction method with the 20-kHz focused ultrasound and reference hot-water extraction approaches. Laboratory analysis and *in situ* applications are both investigated. To minimise energy consumption and liquid handling, we propose simultaneous extraction and derivatisation in a single step. This new analytical strategy directly produces GC-compatible final products, thus reducing the sample preparation time and enhancing analysis performance. For validation, we use samples of the Mukundpura meteorite, one of the most primitive meteorites recently fallen on Earth [24].

## 2 Materials and methods

### 2.1 Materials

**Fig. 1:**
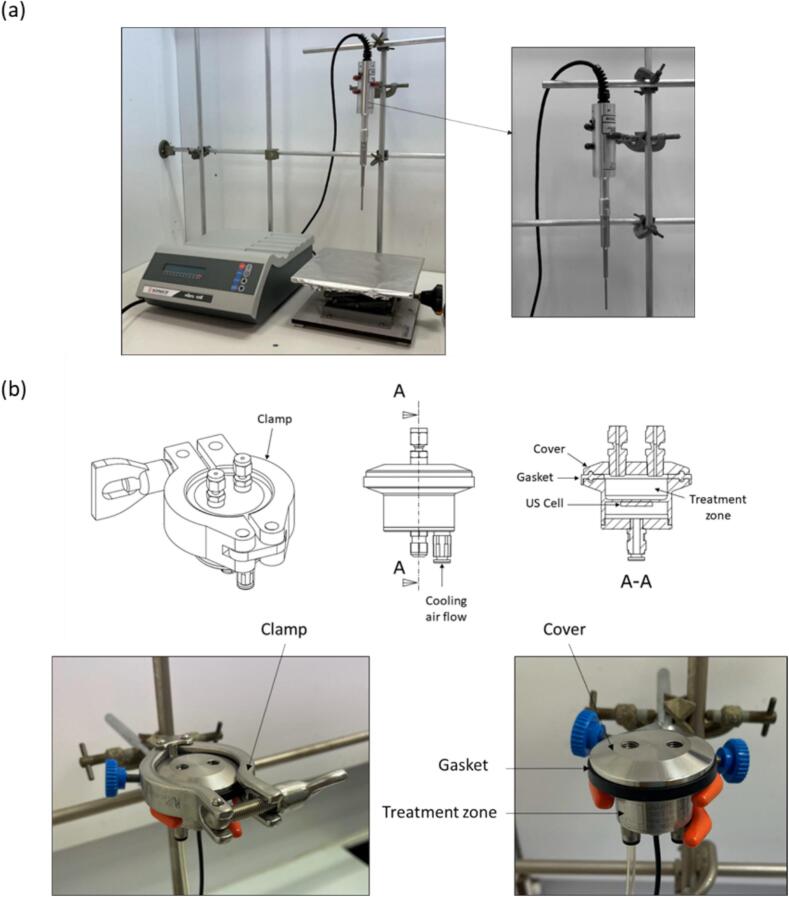
(a) Focused ultrasonic system for extraction, equipped with a 2-mm probe, (b) piezoelectric reactor and its components.

A 20-kHz Sonics Vibra-Cell (130 W, Newtown, USA) focused ultrasound system and a new 2.4 MHz ultrasonic system developed in partnership with SinapTec (Lezennes, France) were used in this work (Fig. 1). Acoustic and electric power for the 20-kHz device was higher (0,35 and 1.255 W/mL respectively) than the one of our custom reactor (0.22 and 0.33 W/mL).

The new system operates at a high frequency of 2.4 MHz in continuous mode. The treatment zone is miniaturised to a diameter of 30 mm, thus concentrating ultrasonic waves in a confined area. A constant flow of air facilitates cell cooling.

### 2.2 Chemicals

Amino acid stock standard solutions were prepared in milliQ water (18.2 MΩ.cm^−1^), starting from individual lyophilised enantiomerically pure compounds. Glycine, *N*-ethylglycine, sarcosine, β-alanine, 2-aminoisobutyric acid, ɣ-aminobutyric acid, 5-aminovaleric acid, l-alanine, l-valine, l-leucine, l-proline, l-threonine, l-glutamic acid, l-methionine, l-phenylalanine, l-2-aminobutyric acid, l-3-aminoisobutyric acid, l-alloisoleucine, dl-3-aminobutyric acid, d-alanine, d-valine, d-leucine, d-proline, d-threonine, d-glutamic acid, d-methionine, d-phenylalanine, d-2-aminobutyric acid, d-3-aminoisobutyric acid, d-valine, d-norvaline, d-homoserine, d-isoleucine, d-norleucine, d-alloisoleucine, d-glutamic acid, d-methionine, d-proline, and d-phenylalanine were used, all with purities ≥ 97 % (Sigma-Aldrich, Steinheim, Germany). l-norvaline, l-norleucine, l-homoserine, d-leucine, and d-aspartic acid had purities ≥ 99 % (Alfa Aesar, Ward Hill, USA). l-isovaline, d-isovaline, d-serine, d-threonine, had purities ≥ 97 % (Acros Organics, Geel, Belgium). l-β-leucine and d-β-leucine exhibited purities ≥ 97 % (Chem-Impex, Wood Dale USA). The purities of the other chemicals were as follows: Methyl chloroformate (MCF) ≥ 99 %; ethyl chloroformate (ECF) ≥ 97 %; butyl chloroformate (BCF) ≥ 98 %; isobutyl chloroformate (IBCF) ≥ 98 %; pyridine, methanol (MeOH), and trifluoroacetic acid (TFA) ≥ 98 % (Sigma-Aldrich); chloroform ≥ 99 % (Acros Organics). Internal standards were *n*-pentadecane (≥ 99 %) for GC–MS/MS and l −alanine-1-^13^C (≥ 99 %) for ultra-performance liquid chromatography–mass spectrometry (UPLC-MS/MS), both purchased from Sigma-Aldrich.

### 2.3 Mineral matrices

Experiments were first conducted on clay-rich model mineral samples. SWy-3 montmorillonite was purchased from the Clay Mineral Society (Chantilly, USA).80 µL solution of 16 amino acids (25 µmol/L) were spiked on 20 mg mineral matrix (20 % montmorillonite and 80 % silica). Two Mukundpura meteorite fragments (India, 2017) were treated at room temperature (20 °C) to remove organics from the surface. To this end, a 2 mL mixture of methanol and water (1:1, v/v) was added to the fragments for 10 min, with gentle mixing for 30 s every 5 min. Subsequently, the fragments were dried for 3 h at 40 °C and crushed separately using an agate mortar. The first crushed fragment was divided into nine samples, each weighing 200 mg, to optimize the 2.4 MHz extraction and to facilitate comparison with the optimal 20 kHz and reference hot-water extraction methods (Table 1). The second fragment was split into two parts: 1 g was used to evaluate the proposed simultaneous extraction and derivatisation method, while the other 1 g was used for comparison with the standard extraction followed by derivatisation method.

**Table 1:** Parameters for 2.4 MHz, 20 kHz, and hot-water extraction approaches for samples from the same Mukundpura meteorite fragment.

**N°**	**Sample name**	**MeOH (%)**	**Time (min)**	**Ultrasound (kHz)**
1	R01-25 %-10 min	25	10	2400
2	R02-25 %-20 min	25	20	2400
3	R03-50 %-10 min	50	10	2400
4	R04-50 %-20 min	50	20	2400
5	R05-75 %-10 min	75	10	2400
6	R06-75 %-20 min	75	20	2400
7	R07-50 %-20 min	50	20	20
8	R08-50 %-10 min	50	10	20
9	R09-100 °C-24 h	0	1440	0

### 2.4 Extraction techniques

**Fig. 2:**
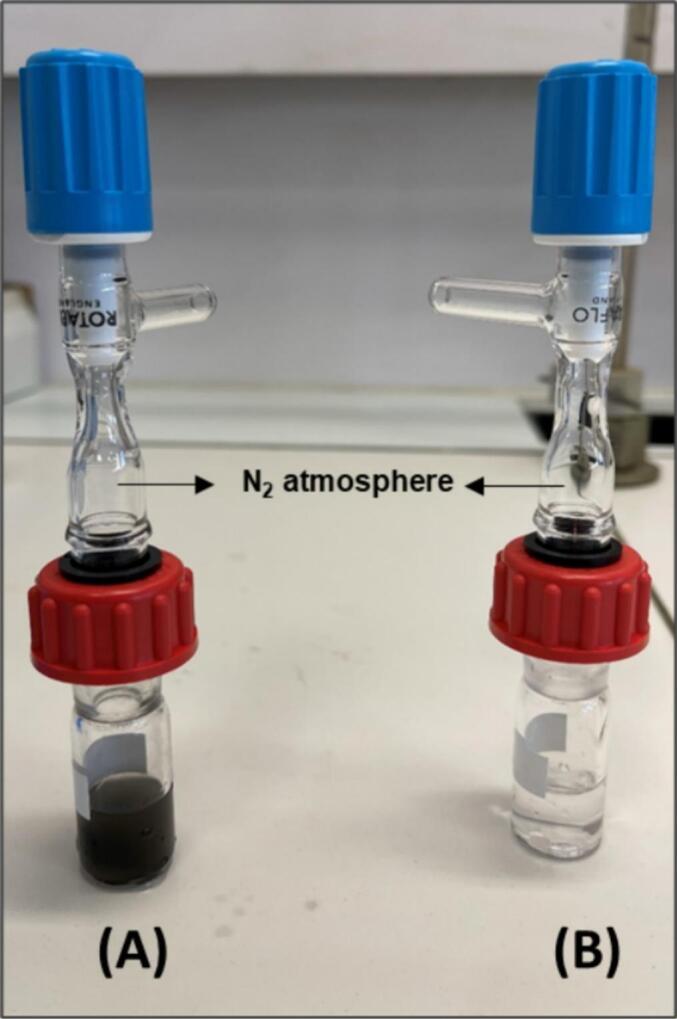
Hot-water extraction vials: (A) meteorite extraction; (B) procedural blank.

All glassware was rinsed with water and methanol, wrapped in aluminium foil, and heated for several hours at 500 °C before use. Extractions were performed using different methods: in the 2.4 MHz reactor, in a 4 mL vial for the 20-kHz extraction, or in a sealed vial under nitrogen for the 24-h 100 °C water extraction (Fig. 2) [25]. For ultrasonic extraction, a methanol and water mixture (2 mL/200 mg) was added. Extractions were performed three times, and the solvent was replaced after each extraction. After centrifugation (10 min at 15000 rpm), extracts were analysed through UPLC-MS/MS for laboratory validation and GC–MS/MS for *in situ* application.

### 2.5 Extraction-derivatisation

The following materials were used in this process: 1 mL water, 1 mL methanol, 200 µL pyridine, and 200 µL methyl chloroformate. The reactor was immediately sealed, and ultrasound waves were applied for 10 min. Extracts were directly analysed through GC–MS/MS after liquid/liquid extraction in 100 µL chloroform. Additionally, 5 µL of the internal standard pentadecane were added before the analysis. The same solvent and reagent volumes were used for direct comparison of the two-step extraction followed by derivatisation method.

### 2.6 Analysis

#### 2.6.1 UPLC-MS/MS

UPLC-MS/MS analysis was performed using a Shimadzu Nexera X2 UPLC–8050 triple quadrupole mass spectrometry system (Shimadzu, Kyoto, Japan). Amino acid enantiomers were separated on a CROWNPAK CR(+) chiral column (150 mm × 4.0 mm, 5 µm film thickness, Chiral Technologies Europe, Illkirch-Graffenstaden, France) equipped with a CROWNPAK CR guard column (10 mm × 4.0 mm, 5 µm film thickness, Chiral Technologies). A water/methanol (95:5, v/v) mixture with 0.5 % TFA mobile phase was supplied at a constant flow of 650 µL.min^−1^. Additionally, 5 µL of the resulting solution was injected. The retention time and multiple reaction monitoring (MRM) transitions are detailed in Table S1.

#### 2.6.2 GC–MS/MS

Extracts were analysed using a GC-triple quadrupole system TQ7010 (Agilent Technologies, Santa Clara, CA, USA). Compounds were separated on a chiral CP-Chirasil-Dex capillary column (25 m × 0.25 mm, 0.25 µm film thickness, Agilent Technologies). A volume of 1 µL was injected, and helium (≥ 99.9999 % purity, Air liquide, Paris, France) flow was set as 1 mL.min^−1^ with a split ratio of 20:1. The oven temperature was set as 100 °C for 5 min and raised to 128 °C at a rate of 3 °C·min^−1^. Subsequently, the temperature was increased to 137 °C at a rate of 1 °C·min^−1^ and then to 190 °C at 3 °C·min^−1^ to be finally maintained for 10 min. MRM enabled precise identification and quantification. Retention time values and MRM transitions are detailed in Table S2.

## 3 Results and discussion

### 3.1 Laboratory validation

**Fig. 3:**
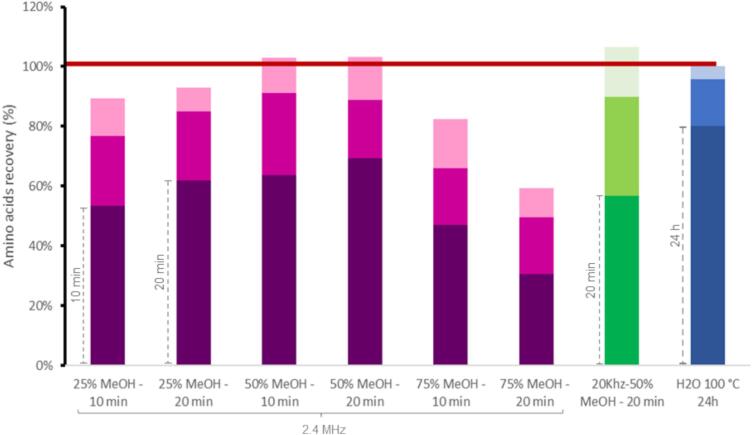
Recovery of Mukundpura amino acids: sum of three sequential 2.4 MHz and 20 kHz ultrasound-assisted extractions compared with three 24 h hot-water extractions set at 100 %.

Clay minerals, recently found in asteroid Ryugu. are in the center of numerous studies on exobiology and the origin of life [26,27]. Supposed to adsorb and preserve organic molecules, these minerals pose significant challenges for organics extraction as shown by previous studies requiring strong oxidative conditions needed to fully remove associated organics [14,28,29]. To validate the efficacy of the 2.4 MHz reactor, ultrasound-assisted extraction was thus performed on clays samples (Fig. S1) and meteorite fragments (Fig. 3). The results were compared with those of the reference hot-water extraction approach. As already stated in previous studies, the efficiency of ultrasonic aqueous extraction of amino acids is improved by the addition of methanol. Ultrasonic extraction at 20-kHz yielded comparable results to hot-water extraction for meteorite samples but prolonged exposure to ultrasound can lea to the degradation of organic molecules such as amino acids [29]. Three methanol and water mixtures (25 %, 50 % and 75 % MeOH) and two durations (10 min and 20 min) were then selected [14,29].

We performed three extractions on each clay samples and analysed each extract through UPLC-MS/MS. For rich clay samples, 2.4 MHz was at least as efficient as 20 kHz one except for 75 % MeOH (Fig. S1). Subsequently, we performed three extractions on each of the eight Mukundpura samples (from MKI-R01 to MKI-R08) and analysed each extract through UPLC-MS/MS (Fig. 3).

After three extractions, the quantity of identified amino acids remained consistent across all configurations. Our results highlighted the necessity of multiple extractions, as amino acids were still recovered in the two additional extractions, even for the reference hot-water method, typically performed once [30]. Additionally, the ultrasonic extracts were similar, except for those with the highest methanol content (75 % MeOH). We attributed this discrepancy of results to the reduced solubility of the targets in methanol. Consistent with observations from prior studies based on 20-kHz focused ultrasonic devices, extraction durations of 10 to 20 min with 50 % MeOH proved optimal for quantitative analysis [29]. The 2.4 MHz reactor yielded qualitative and quantitative recoveries in 30 min (103 %) compared with the three-day duration required for hot-water extraction. The 2.4 MHz extraction was thus as effective as the reference hot-water extraction, despite significantly reduced energy and time consumption.

Additionally, the use of ultrasonic devices with reduced time would mitigate the risk of degradation of native molecules. For laboratory experiments both ultrasonic extractions yielded similarly efficient results whereas, for *in situ* a single *in situ* extraction, 2.4 MHz extraction gave the highest recovery.

### 3.2 *In situ* application

#### 3.2.1 Optimal extraction parameters

Considering energy consumption and the volume of liquids to be handled under space constraints, a single extraction approach is preferable.

**Fig. 4:**
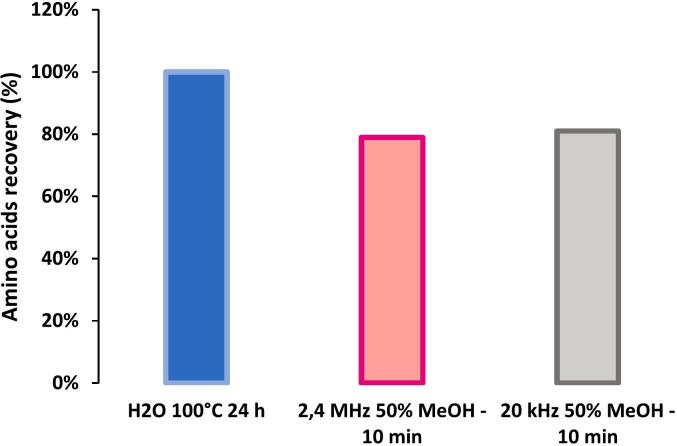
Amino acid recovery for a single 10-min ultrasounds-assisted extraction at 2.4 MHz and 20 kHz compared with 24 h hot-water extraction set as100% recovery.

For *in situ* purposes, shorter durations are generally preferred owing to operational constraints. Thus, in this study, a 10-min duration was selected for Mukundpura analysis, as it yielded a recovery rate of more than 79 % of 24 h hot water recovery (Fig. 4). In comparison, the 10-min 50 % MeOH 20-kHz extraction, previously developed for space applications, achieved an 81 % of 24 h hot water recovery of amino acids.

Notably, if glycine is to be detected, 20-min experiments should be conducted, as glycine was at its limit of quantification (LOQ) with a 10-min extraction (Table 2). In fact, glycine was only quantified in the second and third extractions in 2.4-MHz 10-min experiments but was promptly detected when the 20 kHz 20-min duration was selected [29].The concentration of sarcosine, β-alanine, 2-aminobutyric acid, and β-leucine were lower in ultrasound experiments (ranging from a factor of 2 to 5 compared with hot-water extraction). However, aspartic acid could be extracted only through the ultrasonic method.

**Table 2:** Concentrations of amino acids from Mukundpura extracts (nmol/g).

**Compounds**	**H_2_O 24 h 100 °C R-08**	**20 kHz − 10 min H_2_O:MeOH (1:1, v/v) R-07**	**2.4 MHz − 10 min H_2_O:MeOH (1:1, v/v) R-03**
Glycine	25.5	<LOQ	<LOQ
Sarcosine	3.28	8.13	6.34
D-alanine	13.7	11.4	11.03
β-alanine	14.72	3.62	5.2
L-alanine			
2-aminoisobutyric acid	41.1	56.1	38.42
D-2-aminobutyric acid			
DL-3-aminobutyric acid			
L-2-aminobutyric acid	25.77	5.74	17.7
D-3-aminoisobutyric acid	9.38	16	17.7
L-3-aminoisobutyric acid			
ɣ-aminobutyric acid			
DL-serine	6.04	2.62	2.8
DL-proline*	3.97	2.2	2.61
D-isovaline	15.25	15.5	12.76
L-isovaline			
D-valine			
l-valine	9.45	3.11	3.48
D-norvaline	n.d	n.d	n.d
5-aminovaleric acid	3.46	3.64	3.94
L-norvaline	2.98	2.58	2.22
D-β-leucine	15.84	5.7	8.56
L-β-leucine	n.d	n.d	n.d
D-alloisoleucine	n.d	n.d	n.d
D-isoleucine	4.51	3.04	3.17
L-alloisoleucine	13.1	9.28	16.04
L-isoleucine	8.85	11.9	11.85
D-leucine	5.5	6.57	4.94
D-norleucine	3.27	4.2	2.62
L-leucine	8.98	7.96	7.91
L-norleucine	6.5	3.61	6.09
D-aspartic acid	n.d	4.02	3.86
L-aspartic acid	n.d	2.99	3.31
D-glutamic acid	n.d	n.d	n.d
L-glutamic acid	3.92	3.26	4.05

*: confirmed contamination; nd: not detected; < LOQ: inferior to the limit of quantification

#### 3.2.2 Optimal derivatisation parameters

To better mimic *in situ* analysis, we performed gas chromatography for the following experiments. Derivatisation is essential for amino acids to render them suitable for gas chromatographic analysis. Derivatizing agents must be selected to adhere to both space and extraction constraints. Alkyl chloroformates in aqueous alcohol-pyridine media rapidly (within a few seconds) convert amino acids into derivatives suitable for gas chromatography analysis [31,32]. In addition to their intrinsic advantages, chloroformates enable enantiomeric separation of amino acids.

Thus, four combinations of chloroformate agents and methanol were tested to determine the optimal chiral separation (Tables S2–S5). Table S6 summarises the limit of detection for each configuration. Enantiomeric analyses were performed for 27 amino acids using CP-Chirasil-Dex and Chirasil-L-Val capillary columns. MCF/MeOH derivatisation was selected considering the numbers of identified amino acids and enantiomeric separations. Although Chirasil- L −Val column enabled better enantiomeric separations and limits of detection for certain compounds, the CP-Chirasil-Dex column was selected for its significantly lower background noise, which facilitated trace-level molecule detection.

#### 3.2.3 One-step extraction-derivatisation method

This strategy used common reagents (water and methanol), which served as solvents for ultrasound-assisted extraction and chloroformate derivatisation. All identified amino acids in each extract are outlined in Table 3.

**Table 3:** Mukundpura amino acids recovered by ultrasound-assisted extraction followed by derivatisation (two steps) and simultaneous extraction-derivatisation (one-step) methods and relative quantification (nmol/g of meteorite**)**.

**Compound**	**Two steps**	**One-step**
sarcosine	1.18	0.75
d-alanine	2.01	0.87
l-alanine	2.73	1.72
2-aminoisobutyric acid	7.48	1.92
dl-isovaline	3.68	1.5
*n*-ethylglycine	0.17	0.1
d-2-aminobutyric acid	1.48	0.75
l-2-aminobutyric acid	1.83	0.86
l-valine	0.69	0.37
d-valine	0.81	0.33
glycine	53.55	51.98
d-norvaline	0.26	0.1
l-norvaline	0.33	0.13
dl-3-aminobutyric acid	2.18	1.26
d-3-aminoisobutyric acid	nd	nd
l-3-aminoisobutyric acid	nd	nd
d-norleucine	0.1	0.04
l-norleucine	0.14	0.05
d-alloisoleucine	0.6	0.25
l-alloisoleucine	1.16	0.47
d-isoleucine	0.73	0.33
l-isoleucine		
d-leucine	0.18	0.06
l-leucine	0.29	0.22
d-β-leucine	nd	nd
l-β-leucine	nd	nd
d-proline	0.47	0.2
l-proline	0.7	0.52
ɣ-aminobutyric acid	<LOQ	6.46
dl-aspartic acid	3.09	<LOQ
5-amino valeric acid	4.53	<LOQ

nd: not detected; < LOQ: inferior to the limit of quantification

The identified amino acids were consistent with those extracted in prior experiments on Mukundpura meteorite [24,29]. The simultaneous extraction-derivatisation method could successfully recover quantitative yields of derivatised amino acids. When considering all quantified amino acids in the extract, we achieved a recovery rate of 78 % relative to the extraction followed by derivatisation method. This shortened and simplified sample pretreatment presents for the first time a one-step extraction-derivatisation protocol that could enhance the detection of amino acids to compete thermodesorption and thermochemolysis already implemented in space missions.

## 4 Conclusions

This paper introduces a novel ultrasonic reactor for the efficient extraction of amino acids in meteorite samples. Since we obtained similar extraction yields without racemization as the reference 24 h-hot water extraction, we could envisage ultrasounds extraction as a faster and low energetic alternative. Conducting several extractions to achieve the best recoveries is however essential for laboratory experiments, whatever the procedure considered. The optimal parameters for *in situ* analysis were identified, resulting in the selection of a mixture of methanol and water (1:1, v/v) for a 10-min extraction, enabling 80 % recovery compared with the 24 h reference method, while mitigating the risk of degradation of native molecules with prolonged US duration. The novel simultaneous extraction and derivatisation strategy simplify the future automation of the detection process by producing a quantitative recovery and ready-for-analysis organic targets for GC–MS/MS analysis. Further experiments should be conducted for automation for *in situ* as well as future return samples analysis.

## CRediT authorship contribution statement

**Ramzi Timoumi:** Writing – original draft, Investigation. **Rihab Fkiri:** Investigation. **Prince Amaniampong:** Writing – review & editing, Validation, Methodology. **Guillaume Rioland:** Supervision, Funding acquisition. **Brian Gregoire:** Writing – review & editing, Supervision. **Pauline Poinot:** Writing – review & editing, Validation, Supervision, Methodology. **Claude Geffroy-Rodier:** Writing – review & editing, Validation, Supervision, Methodology, Conceptualization.

## Declaration of competing interest

The authors declare that they have no known competing financial interests or personal relationships that could have appeared to influence the work reported in this paper.

## References

[1] Millan M., Teinturier S., Malespin C.A., Bonnet J.Y., Buch A., Dworkin J.P., Eigenbrode J.L., Freissinet C., Glavin D.P., Navarro-González R., Srivastava A., Stern J.C., Sutter B., Szopa C., Williams A.J., Williams R.H., Wong G.M., Johnson S.S., Mahaffy P.R. (2022). Organic molecules revealed in Mars’s bagnold dunes by Curiosity’s derivatization experiment. Nat Astron.

[2] Millan M., Szopa C., Buch A., Coll P., Glavin D.P., Freissinet C., Navarro-Gonzalez R., François P., Coscia D., Bonnet J.Y., Teinturier S., Cabane M., Mahaffy P.R. (2016). In situ analysis of martian regolith with the SAM experiment during the first mars year of the MSL mission: identification of organic molecules by gas chromatography from laboratory measurements. Planet. Space Sci..

[3] Eigenbrode J.L., Summons R.E., Steele A., Freissinet C., Millan M., Navarro-González R., Sutter B., McAdam A.C., Franz H.B., Glavin D.P., Archer P.D., Mahaffy P.R., Conrad P.G., Hurowitz J.A., Grotzinger J.P., Gupta S., Ming D.W., Sumner D.Y., Szopa C., Malespin C., Buch A., Coll P. (2018). Organic matter preserved in 3-billion-year-old mudstones at Gale crater, Mars. Science.

[4] Burton A.S., Berger E.L. (2018). Insights into abiotically-generated amino acid Enantiomeric excesses found in meteorites. Life.

[5] Burton A.S., McLain H., Glavin D.P., Elsila J.E., Davidson J., Miller K.E., Andronikov A.V., Lauretta D., Dworkin J.P. (2015). Amino acid analyses of R and CK chondrites. Meteorit. Planet. Sci..

[6] Cronin J.R., Pizzarello S. (1983). Amino acids in meteorites. Adv. Space Res..

[7] Elsila J.E., Aponte J.C., Blackmond D.G., Burton A.S., Dworkin J.P., Glavin D.P. (2016). Meteoritic amino acids: diversity in compositions reflects Parent body histories. ACS Cent. Sci..

[8] Oba Y., Koga T., Takano Y., Ogawa N.O., Ohkouchi N., Sasaki K., Sato H., Glavin D.P., Dworkin J.P., Naraoka H., Tachibana S., Yurimoto H., Nakamura T., Noguchi T., Okazaki R., Yabuta H., Sakamoto K., Yada T., Nishimura M., Nakato A., Miyazaki A., Yogata K., Abe M., Okada T., Usui T., Yoshikawa M., Saiki T., Tanaka S., Terui F., Nakazawa S., Watanabe S., Tsuda Y. (2023). Uracil in the carbonaceous asteroid (162173) ryugu. Nat Commun.

[9] Naraoka H., Takano Y., Dworkin J.P., Oba Y., Hamase K., Furusho A., Ogawa N.O., Hashiguchi M., Fukushima K., Aoki D., Schmitt-Kopplin P., Aponte J.C., Parker E.T., Glavin D.P., McLain H.L., Elsila J.E., Graham H.V., Eiler J.M., Orthous-Daunay F.-R., Wolters C., Isa J., Vuitton V., Thissen R., Sakai S., Yoshimura T., Koga T., Ohkouchi N., Chikaraishi Y., Sugahara H., Mita H., Furukawa Y., Hertkorn N., Ruf A., Yurimoto H., Nakamura T., Noguchi T., Okazaki R., Yabuta H., Sakamoto K., Tachibana S., Connolly H.C., Lauretta D.S., Abe M., Yada T., Nishimura M., Yogata K., Nakato A., Yoshitake M., Suzuki A., Miyazaki A., Furuya S., Hatakeda K., Soejima H., Hitomi Y., Kumagai K., Usui T., Hayashi T., Yamamoto D., Fukai R., Kitazato K., Sugita S., Namiki N., Arakawa M., Ikeda H., Ishiguro M., Hirata N., Wada K., Ishihara Y., Noguchi R., Morota T., Sakatani N., Matsumoto K., Senshu H., Honda R., Tatsumi E., Yokota Y., Honda C., Michikami T., Matsuoka M., Miura A., Noda H., Yamada T., Yoshihara K., Kawahara K., Ozaki M., Iijima Y., Yano H., Hayakawa M., Iwata T., Tsukizaki R., Sawada H., Hosoda S., Ogawa K., Okamoto C., Hirata N., Shirai K., Shimaki Y., Yamada M., Okada T., Yamamoto Y., Takeuchi H., Fujii A., Takei Y., Yoshikawa K., Mimasu Y., Ono G., Ogawa N., Kikuchi S., Nakazawa S., Terui F., Tanaka S., Saiki T., Yoshikawa M., Watanabe S., Tsuda Y. (2023). Soluble organic molecules in samples of the carbonaceous asteroid (162173) ryugu. Science 379.

[10] Rudraswami N.G., Naik A.K., Tripathi R.P., Bhandari N., Karapurkar S.G., Prasad M.S., Babu E.V.S.S.K., Vijaya Sarathi U.V.R. (2019). chemical, isotopic and amino acid composition of mukundpura CM2.0 (CM1) chondrite: evidence of parent body aqueous alteration. Geosci. Front..

[11] Altwegg K., Balsiger H., Bar-Nun A., Berthelier J.-J., Bieler A., Bochsler P., Briois C., Calmonte U., Combi M.R., Cottin H., De Keyser J., Dhooghe F., Fiethe B., Fuselier S.A., Gasc S., Gombosi T.I., Hansen K.C., Haessig M., Jäckel A., Kopp E., Korth A., Le Roy L., Mall U., Marty B., Mousis O., Owen T., Rème H., Rubin M., Sémon T., Tzou C.-Y., Hunter Waite J., Wurz P. (2016). Prebiotic chemicals—amino acid and phosphorus—in the coma of comet 67P/Churyumov-gerasimenko. Science Advances 2.

[12] Buch A., Sternberg R., Meunier D., Rodier C., Laurent C., Raulin F., Vidal-Madjar C. (2003). Solvent extraction of organic molecules of exobiological interest for in situ analysis of the Martian soil. J. Chromatogr. A.

[13] R. Sternberg, A. Buch, J.J. Correa, P. Chazalnoel, Device for preparing a sample, 2,260,287 A2, n.d.

[14] Timoumi R., François P., Le Postollec A., Dobrijevic M., Grégoire B., Poinot P., Geffroy-Rodier C. (2022). Focused ultrasound extraction versus microwave-assisted extraction for extraterrestrial objects analysis. Anal Bioanal Chem.

[15] Parro V., de Diego-Castilla G., Rodríguez-Manfredi J.A., Rivas L.A., Blanco-López Y., Sebastián E., Romeral J., Compostizo C., Herrero P.L., García-Marín A., Moreno-Paz M., García-Villadangos M., Cruz-Gil P., Peinado V., Martín-Soler J., Pérez-Mercader J., Gómez-Elvira J. (2011). SOLID3: a multiplex antibody Microarray-based optical sensor instrument for in situ life detection in Planetary exploration. Astrobiology.

[16] Buch A., Glavin D.P., Sternberg R., Szopa C., Rodier C., Navarro-González R., Raulin F., Cabane M., Mahaffy P.R. (2006). A new extraction technique for in situ analyses of amino and carboxylic acids on Mars by gas chromatography mass spectrometry. Planet. Space Sci..

[17] David M., Musadji N.-Y., Labanowski J., Sternberg R., Geffroy-Rodier C. (2016). Pilot for validation of online Pretreatments for analyses of organics by gas chromatography–mass spectrometry: application to space Research. Anal. Chem..

[18] Poinot P., Geffroy-Rodier C. (2015). Searching for organic compounds in the universe. TrAC Trends Anal. Chem..

[19] Peer Reviewed: Analyzing a Comet Nucleus by Capillary GC, Anal. Chem. 74 (2002) 481 A-487 A. 10.1021/ac022109q. 12236379

[20] Rodier C., Sternberg R., Szopa C., Buch A., Cabane M., Raulin F. (2005). Search for organics in extraterrestrial environments by in situ gas chromatography analysis. Adv. Space Res..

[21] Fkiri R., Timoumi R., Rioland G., Poinot P., Baron F., Gregoire B., Geffroy-Rodier C. (2023). Gas chromatography fingerprint of Martian amino acids before analysis of return samples. Chemosensors.

[22] Geffroy-Rodier C., Grasset L., Sternberg R., Buch A., Amblès A. (2009). Thermochemolysis in search for organics in extraterrestrial environments. J. Anal. Appl. Pyrolysis.

[23] Buch A., Sternberg R., Szopa C., Freissinet C., Garnier C., Bekri E.J., Rodier C., Navarro-González R., Raulin F., Cabane M., Stambouli M., Glavin D.P., Mahaffy P.R. (2009). Development of a gas chromatography compatible sample processing system (SPS) for the in-situ analysis of refractory organic matter in martian soil: preliminary results. Adv. Space Res..

[24] Pizzarello S., Yarnes C.T. (2018). The soluble organic compounds of the mukundpura meteorite: a new CM chondrite fall. Planet. Space Sci..

[25] Botta O., Glavin D.P., Kminek G., Bada J.L. (2002). Relative amino acid concentrations as a signature for parent body processes of carbonaceous chondrites. Origins Life Evol. Biospheres.

[26] Viennet J.-C., Roskosz M., Nakamura T., Beck P., Baptiste B., Lavina B., Alp E.E., Hu M.Y., Zhao J., Gounelle M., Brunetto R., Yurimoto H., Noguchi T., Okazaki R., Yabuta H., Naraoka H., Sakamoto K., Tachibana S., Yada T., Nishimura M., Nakato A., Miyazaki A., Yogata K., Abe M., Okada T., Usui T., Yoshikawa M., Saiki T., Tanaka S., Terui F., Nakazawa S., Watanabe S.-I., Tsuda Y. (2023). Interaction between clay minerals and organics in asteroid ryugu. Geochem. Persp. Let..

[27] (Theo) Kloprogge J.T., Hartman H. (2022). Clays and the origin of life: the Experiments. Life.

[28] Samanta A., Bera M.K., Sreenivasan S.P., Sarkar A. (2022). Comparison of Pretreatment methods for organic-matter removal and their effects on the hydrogen isotope (δ2H) composition of kaolinite. Clay Clay Miner..

[29] Timoumi R., Amaniampong P., Le Postollec A., Dobrijevic M., Rioland G., Gregoire B., Poinot P., Rodier C.G. (2024). Ultrasound assisted extraction of amino acids and nucleobases from clay minerals and astrobiological samples. Ultrason. Sonochem..

[30] Simkus D.N., Aponte J.C., Elsila J.E., Parker E.T., Glavin D.P., Dworkin J.P. (2019). Methodologies for analyzing soluble organic compounds in extraterrestrial samples: amino acids. Amines, Monocarboxylic Acids, Aldehydes, and Ketones, Life.

[31] Husek P. (1998). Chloroformates in gas chromatography as general purpose derivatizing agents. J Chromatogr B Biomed Sci Appl.

[32] Rodier C., Laurent C., Szopa C., Sternberg R., Raulin F. (2002). Chirality and the origin of life: in situ enantiomeric separation for future space missions. Chirality.

