# Supplementary material for: New ultrasound-assisted microreactor for extracting extraterrestrial biomolecules
Source: Ultrason Sonochem. 2025 Jun 3;119:107416. doi: 10.1016/j.ultsonch.2025.107416 (PMC12171622; doi:10.1016/j.ultsonch.2025.107416)
Supplement: Supplementary Data 1 [file mmc1.docx]

**Supplementary data:** **New ultrasound-assisted microreactor for extracting extraterrestrial biomolecules**

Ramzi Timoumi ^a^, Rihab Fkiri ^a^, Prince Amaniampong ^a^, Guillaume Rioland ^b^, Brian Gregoire ^a^, Pauline Poinot ^a^, Claude Geffroy Rodier  ^a,*^

**Affiliations**

^a^ UMR CNRS 7285, Institut de Chimie des Milieux et Matériaux de Poitiers (IC2MP), University of Poitiers, 4 rue Michel-Brunet, TSA 51106, 86073 Poitiers Cedex 9, France

^b^ Centre National d’Etudes Spatiales (CNES), Service Laboratoires & Expertise, 18 Avenue Edouard Belin, CEDEX 9, 61401 Toulouse, France

**^*^ Corresponding Author**

Postal address : UMR CNRS 7285, Institut de Chimie des Milieux et Matériaux de Poitiers (IC2MP), E.BiCoM Team, 4 rue Michel-Brunet, TSA 51106, 86073 Poitiers cedex 9, France

Phone: +33-5-49-45-35-90

E-mail address: [claude.geffroy@univ-poitiers.fr](mailto:claude.geffroy@univ-poitiers.fr)

**Fig. S1**. Variation of total extracted amino acid amounts from doped clays depending on solvent and time conditions for 2.4MHz and 20 kHz frequencies. (∑A/A_IS_ total sum of area to internal standard area ratios of each molecule). Direct injection of extracts in UPLC/MS/MS

**Table S1**

Amino acids analyzed by UPLC-MS/MS (MRM transitions, retention times, LODs and LOQs).[1]

| Compound | MRM | | Retention times (min) | LOD (mol.L^-1^) | LOQ (mol.L^-1^) |
| --- | --- | --- | --- | --- | --- |
|  | Transitions | CE (eV) |  |  |  |
| glycine | 76.15 > 30.3 | 18.5 | 2.3 | 2,86.10^-7^ | 4,51.10^-7^ |
| sarcosine  d-alanine  β-alanine  l-alanine | 90.35 > 44.15  90.35 > 45.2 | 15  28.2 | 1.99  2.19  2.52  2.52 | 1,79.10^-7^  2,26.10^-7^  3,45.10^-7^  3,45.10^-7^ | 3,32.10^-7^  3,62.10^-7^  8,18.10^-7^  8,18.10^-7^ |
| n-ethylglycine  2AIBA*  d-2ABA*  dl-3ABA  l-2ABA  d-3AIBA  l-3AIBA  GABA* | 104.25 > 43.1  104.25 > 41.15  104.25 > 42.3  104.25 > 45.1 | 30  25  43  36 | 2.07  2.3  2.3  2.3  2.63  2.85  2.85  2.85 | 1,05.10^-7^  1,26.10^-7^  1,26.10^-7^  1,26.10^-7^  9,17.10^-8^  1,32.10^-7^  1,32.10^-7^  1,32.10^-7^ | 2,52.10^-7^  2,63.10^-7^  2,63.10^-7^  2,63.10^-7^  1,76.10^-7^  3,01.10^-7^  3,01.10^-7^  3,01.10^-7^ |
| d-serine  l-serine | 106.15 > 60.1  106.15 > 88.3 | 12.9  14.9 | 2.06  2.22 | 1,31.10^-7^ | 1,99.10^-7^ |
| dl-proline | 116.35 > 70.35 | 16 | 2.13 | 9,78.10^-8^ | 1,91.10^-7^ |
| dl-isovaline  d-valine  l-valine  d-norvaline  5-aminovaleric acid  l-norvaline | 118.3 > 72.15  118.3 > 55.35 | 12.9  22.2 | 2.56  2.56  2.8  3.03  3.3  4.03 | 6,91.10^-8^  6,91.10^-8^  1,22.10^-7^  8,58.10^-8^  1,02.10^-7^  9,38.10^-8^ | 1,26.10^-7^  1,26.10^-7^  2,7.10^-7^  1,62.10^-7^  2,01.10^-7^  1,68.10^-7^ |
| d-threonine  d-homoserine  l-threonine  l-homoserine | 120.35 > 74.05  120.35 > 56.25 | 11.6  16.3 | 2.06  2.06  2.25  2.33 | 1,57.10^-7^  1,57.10^-7^  7,15.10^-8^  1,10^-7^ | 3,58.10^-7^  3,58.10^-7^  1,78.10^-7^  2,41.10^-7^ |
| dl-β-leucine | 132.3 > 86.15  132.3 > 44.15 | 11.3  22.8 | 2.84 | 8,07.10^-8^ | 1,56.10^-7^ |
| d-alloisoleucine  d-isoleucine  l-alloisoleucine  l-isoleucine  d-leucine  d-norleucine  l-leucine  l-norleucine | 132.3> 41.1 | 29.6 | 3.92  4.07  4.19  4.54  4.73  5.43  6.78  7.79 | 9,2.10^-8^  7,21.10^-8^  8,34.10^-8^  8,51.10^-8^  8,57.10^-8^  7,69.10^-8^  7,93.10^-8^  6,41.10^-8^ | 2,23.10^-7^  1,69.10^-7^  1,56.10^-7^  1,67.10^-7^  1,62.10^-7^  1,48.10^-7^  1,36.10^-7^  1,07.10^-7^ |
| d-aspartic acid  l-aspartic acid | 134.3 > 74.2  134.3 > 88.1 | 15.9  11.3 | 2.17  2.37 | 9,01.10^-8^  9,32.10^-8^ | 2,17.10^-7^  1,94.10^-7^ |
| d-lysine  l-lysine | 147.3 > 84.25  147.3 > 130.2 | 19  13.2 | 2.49  2.69 | 7,64.10^-8^  9,71.10^-8^ | 1,58.10^-7^  2,37.10^-7^ |
| d-glutamic acid  l-glutamic acid | 148.35 > 84.1  148.35 > 56.15 | 18.2  28.4 | 2.26  3.3 | 1,77.10^-7^  1,23.10^-7^ | 2,92.10^-7^  2,38.10^-7^ |
| d-methionine  l-methionine | 150.3 > 56.2  150.3 > 104.2 | 17  12.9 | 3.61  5.46 | 2,19.10^-8^  7,74.10^-8^ | 1,16.10^-7^  2,39.10^-7^ |
| dl-histidine | 156.35 > 110.1  156.35 > 93.25 | 16  25.2 | 2.14 | 8,88.10^-8^ | 1,59.10^-7^ |
| d-phenylalanine  l-phenylalanine | 166.3 > 120.35  166.3 > 103.1 | 14.9  25.5 | 7.86  9.8 | 4,86.10^-8^  5,56.10^-8^ | 9,71.10^-8^  1,04.10^-7^ |
| d-tyrosine  l-tyrosine | 182.3 > 91.15  182.3 > 55.35 | 29.1  13.9 | 4.92  6.24 | 1,8.10^-7^  2,05.10^-7^ | 2,17.10^-7^  3,05.10^-7^ |
| alanine ^13^C | 91.2 > 44.3  91.2 > 46.25  91.2 > 29.03 | -14  -36  -38 | 2.47 |  |  |

*AIBA 2-aminoisobutyric acid, 2ABA 2-aminobutyric acid, GABA ɣ-aminobutyric acid.

**Table S2**

MCF/MeOH derivatives analysed by GC-MS/MS (MRM transitions and retention times)

| N° | Compound | Chirasil-Dex | | Retention times (min) | Compound | Chirasil-L-Val | | Retention times (min) |
| --- | --- | --- | --- | --- | --- | --- | --- | --- |
|  |  | Transitions | CE (eV) |  |  | Transitions | CE (eV) |  |
| 1 | sarcosine | **102 > 58.1**  102 > 42.1 | 5  30 | 10.82 | sarcosine | **161 > 102.1**  102 > 42.1 | 5  30 | 4.32 |
| 2 | D-alanine | **102 > 58.1**  70 > 42.1 | 5  10 | 11.24 | N-ethylglycine | **116 > 59**  116 > 43.8 | 10  10 | 5.8 |
| 3 | 2-AiBA | **116 > 72.1**  84 > 56 | 10  10 | 11.33 | D-alanine | **102 > 58.1**  70 > 42.1 | 5  10 | 6.32 |
| 4 | L-alanine | **102 > 58.1**  70 > 42.1 | 5  10 | 11.53 | 2-AiBA | **116 > 72.1**  84 > 56 | 10  10 | 6.33 |
| 5 | DL-isovaline | **130 > 55.2**  98 > 55.1 | 15  5 | 12.06 | L-alanine | **102 > 58.1**  70 > 42.1 | 5  10 | 6.73 |
| 6 | N-ethylglycine | **116 > 59**  116 > 43.8 | 10  10 | 12.36 | DL-isovaline | **130 > 87.1**  128 > 56.1 | 5  10 | 6.82 |
| 7 | D-2ABA | **116 > 72.1**  84 > 56 | 5  10 | 13.41 | glycine | **88 > 59**  88 > 43.8 | 10  5 | 7.38 |
| 8 | L-2ABA | **116 > 72.1**  84 > 56 | 5  10 | 13.5 | DL-2ABA | **116 > 72.1**  84 > 56 | 5  10 | 9.19 |
| 9 | L-valine | **130 > 98.1**  98 > 55.1 | 5  5 | 13.77 | D-valine | **130 > 98.1**  98 > 55.1 | 5  5 | 9.68 |
| 10 | D-valine | **130 > 98.1**  98 > 55.1 | 5  5 | 14.03 | β-alanine | **98 > 70**  74 > 43 | 5  10 | 9.72 |
| 11 | glycine | **88 > 59**  88 > 43.8 | 10  5 | 14.97 | L-valine | **130 > 98.1**  98 > 55.1 | 5  5 | 10.43 |
| 12 | DL-3ABA | **102 > 58.1**  70 > 42.1 | 5  10 | 15.99 | D-3AIBA | **88 > 57**  88 > 43.8 | 5  5 | 10.63 |
| 13 | D-norvaline | **130 > 88.1**  98 > 55.1 | 5  10 | 16.13 | L-3AIBA | **88 > 57**  88 > 43.8 | 5  5 | 10.79 |
| 14 | D-alloisoleucine | **144 > 88.1**  144 > 69.1 | 5  10 | 16.23 | DL-3ABA | **102 > 58.1**  70 > 42.1 | 5  10 | 11.25 |
| 15 | L-alloisoleucine | **144 > 88.1**  144 > 69.1 | 5  10 | 16.31 | D-proline | **128 > 84.1**  128 > 42.1 | 5  15 | 12.05 |
| 16 | L-norvaline | **130 > 88.1**  98 > 55 | 5  10 | 16.44 | L-proline | **128 > 84.1**  128 > 42.1 | 5  15 | 12.15 |
| 17 | DL-isoleucine | **144 > 88.1**  115 > 83 | 5  5 | 16.99 | D-norvaline | **130 > 88.1**  98 > 55.1 | 5  10 | 12.28 |
| 18 | L-leucine* | **144 > 88.1**  112 > 69 | 5  5 | 17.74 | D-alloisoleucine | **144 > 88.1**  144 > 69.1 | 5  10 | 12.86 |
| 19 | β-alanine* | **98 > 70**  74 > 43 | 5  10 | 17.77 | L-norvaline | **130 > 88.1**  98 > 55.1 | 5  10 | 13.04 |
| 20 | D-leucine | **144 > 88.1**  112 > 69 | 5  5 | 17.96 | pentadecane | **71 > 43.1**  57 > 41.1 | 5  10 | 13.08 |
| 21 | DL-3AIBA | **88 > 57**  88 > 43.8 | 5  5 | 18.76 | D-isoleucine | **144 > 88.1**  115> 88.1 | 5  5 | 13.37 |
| 22 | DL-β-leucine | **128 > 96**  74 > 43.1 | 5  10 | 19.56 | L-alloisoleucine | **144 > 88.1**  144 > 69.1 | 5  10 | 13.4 |
| 23 | D-norleucine | **144 > 88.1**  112 > 41.1 | 5  20 | 20.09 | L-isoleucine | **144 > 88.1**  115> 88.1 | 5  5 | 13.79 |
| 24 | L-norleucine* | **144 > 88.1**  112 > 41.1 | 5  20 | 20.45 | D-leucine | **144 > 88.1**  112> 69.1 | 5  5 | 14.54 |
| 25 | D-proline* | **128 > 84.1**  128 > 42.1 | 5  15 | 20.47 | L-Leucine | **144 > 88.1**  112> 69.1 | 5  5 | 14.99 |
| 26 | L-proline | **128 > 84.1**  128 > 42.1 | 5  15 | 20.92 | D-norleucine | **144 > 88.1**  112> 41.1 | 5  20 | 15.65 |
| 27 | pentadecane | **71 > 43.1**  57 > 41.1 | 5  10 | 24.14 | L-norleucine | **144 > 88.1**  112> 41.1 | 5  20 | 16.26 |
| 28 | GABA | **112 > 69**  88 > 43.8 | 5  5 | 26.1 | D-β-leucine | **128 > 96**  74> 43.1 | 5  10 | 16.38 |
| 29 | DL-aspartic acid | **160 > 128.1**  128 > 96.1 | 0  5 | 27.45 | L-β-leucine | **128 > 96**  74> 43.1 | 5  10 | 16.64 |
| 30 | L-threonine | **115 > 83**  115 > 59 | 5  10 | 27.52 | GABA | **112 > 69**  88 > 43.8 | 5  5 | 16.97 |
| 31 | D-threonine | **115 > 83.1**  115 > 59.1 | 5  10 | 27.66 | L-aspartic acid | **160 > 128.1**  128 > 96.1 | 0  5 | 18.97 |
| 32 | D-homoserine | **115 > 100.1**  100 > 56 | 0  5 | 30.13 | D-aspartic acid | **160 > 128.1**  128 > 96.1 | 0  5 | 19.25 |
| 33 | L-homoserine | **115 > 100.1**  100 > 56 | 0  5 | 30.36 | L-threonine | **115 > 83**  115 > 59 | 5  10 | 23.26 |
| 34 | 5-amino valeric acid | **114 > 86.1**  114 > 43.2 | 5  15 | 31.09 | D-threonine | **115 > 83**  115 > 59 | 5  10 | 23.55 |
| 35 | DL-glutamic acid | **142 > 114.2**  114 > 82 | 0  5 | 32.99 | D-methionine | **147> 115.1**  115 > 83 | 5  10 | 24.88 |
| 36 | DL-methionine | **147 > 115.1**  115 > 83 | 5  5 | 33.29 | L-methionine | **147> 115.1**  115 > 83 | 5  10 | 25.38 |
| 37 | D-isoserine | **96 > 40.1**  88 > 43.8 | 20  5 | 34.13 | D-phenylalanine | **162> 77.1**  91 > 65 | 45  15 | 27.5 |
| 38 | L-isoserine | **96 > 40.1**  88 > 43.8 | 20  5 | 34.23 | L-phenylalanine | **162> 77.1**  91 > 65 | 45  15 | 27.87 |
| 39 | DL-phenylalanine | **162 > 77.1**  91 > 65 | 45  15 | 37.66 |  |  |  |  |

**Table S3**

ECF/MeOH derivatives analysed by GC-MS/MS (MRM transitions and retention times)

| N° | Compound | Chirasil-Dex | | Retention times (min) | Compound | Chirasil-L-Val | | Retention times (min) |
| --- | --- | --- | --- | --- | --- | --- | --- | --- |
|  |  | Transitions | CE (eV) |  |  | Transitions | CE (eV) |  |
| 1 | sarcosine | **116 > 72**  116 > 44.1 | 5  10 | 15.38 | sarcosine | **116 > 72**  116 > 44.1 | 5  10 | 6.7 |
| 2 | 2-AIBA | **130 > 58.1**  84 > 56 | 10  10 | 16.15 | N-ethylglycine | **189 > 116.1**  86 > 58 | 10  10 | 7.34 |
| 3 | D-alanine | **116 > 44.1**  70 > 42.1 | 10  10 | 16.38 | D-alanine | **116 > 44.1**  70 > 42.1 | 10  10 | 7.85 |
| 4 | L-alanine | **116 > 44.1**  70 > 42.1 | 10  10 | 16.69 | 2-AiBA | **130 > 58.1**  84 > 56 | 10  10 | 7.92 |
| 5 | N-ethylglycine | **189 > 116.1**  86 > 58 | 0  5 | 16.69 | L-alanine | **116 > 44.1**  70 > 42.1 | 0  5 | 8.46 |
| 6 | DL-isovaline | **144 > 72**  98 > 55.1 | 10  10 | 17.02 | DL-isovaline | **144 > 72**  98 > 55.1 | 10  10 | 8.61 |
| 7 | DL-2ABA | **84 > 56**  84 > 41.1 | 10  5 | 18.32 | glycine | **102 > 74**  88 > 44.1 | 10  5 | 8.93 |
| 8 | D-valine | **115 > 83**  98 > 55 | 5  5 | 18.63 | DL-2ABA | **84 > 56**  84 > 41.1 | 5  5 | 10.05 |
| 9 | L-valine | **115 > 83**  98 > 55 | 5  5 | 19.01 | D-valine | **115 > 83**  98 > 55 | 5  5 | 10.44 |
| 10 | glycine | **102 > 74**  88 > 44.1 | 5  5 | 20.44 | β-alanine | **98 > 70**  74 > 43 | 5  5 | 10.53 |
| 11 | DL-norvaline | **98 > 70**  98 > 55.1 | 10  10 | 20.99 | L-valine | **115 > 83**  98 > 55 | 10  10 | 11,18 |
| 12 | D-alloisoleucine | **158 > 102.1**  112 > 69.1 | 5  5 | 21.03 | D-3AIBA | **112 > 84**  88 > 57 | 5  5 | 11,23 |
| 13 | L-alloisoleucine | **158 > 102.1**  112 > 69.1 | 5  5 | 21.26 | L-3AIBA | **112 > 84**  88 > 57 | 5  5 | 11,38 |
| 14 | D-isoleucine | **115 > 83**  115 > 45 | 5  25 | 21.65 | DL-3ABA | **115 > 83**  115 > 45 | 5  25 | 11,89 |
| 15 | L-isoleucine | **115 > 83**  115 > 45 | 5  25 | 21.83 | D-proline | **142 > 98.1**  142 > 70.1 | 5  25 | 12,73 |
| 16 | D-leucine | **112 > 69**  112 > 41.1 | 5  25 | 22.2 | L-proline | **142 > 98.1**  142 > 70.1 | 5  25 | 12,82 |
| 17 | D-3AIBA | **112 > 84**  88 > 57 | 0  5 | 22.39 | pentadecane | **71 > 43.1**  57 > 41.1 | 0  5 | 13,08 |
| 18 | L-leucine* | **112 > 69**  112 > 41.1 | 5  25 | 22.52 | D-norvaline | **98 > 70**  98 > 55.1 | 0  5 | 13,07 |
| 19 | β-alanine* | **98 > 70**  74 > 43 | 5  10 | 22.57 | D-alloisoleucine | **158 > 102.1**  112 > 69.1 | 5  25 | 13,51 |
| 20 | L-3AIBA | **112 > 84**  88 > 57 | 0  5 | 23.08 | L-norvaline | **98 > 70**  98 > 55.1 | 5  10 | 13,67 |
| 21 | DL-β-leucine | **174 > 60**  74 > 43.1 | 10  10 | 23.49 | D-isoleucine | **115 > 83**  115 > 45 | 10  10 | 14,11 |
| 22 | D-proline | **142 > 98.1**  142 > 70.1 | 5  10 | 23.53 | L-alloisoleucine | **158 > 102.1**  112 > 69.1 | 5  10 | 14,24 |
| 23 | L-proline | **142 > 98.1**  142 > 70.1 | 5  10 | 23.93 | L-isoleucine | **115 > 83**  115 > 45 | 5  10 | 14,62 |
| 24 | DL-norleucine | **112 > 69**  69 > 41.1 | 5  5 | 24.08 | D-leucine | **112 > 69**  112 > 41.1 | 5  5 | 15,29 |
| 25 | pentadecane | **71 > 43.1**  57 > 41.1 | 5  10 | 24.24 | L-leucine | **112 > 69**  112 > 41.1 | 5  10 | 15,67 |
| 26 | GABA | **100 > 44**  74 > 43 | 25  5 | 27.42 | D-norleucine | **112 > 69**  69 > 41.1 | 25  5 | 16,12 |
| 27 | D-threonine | **161 > 129.1**  129 > 101.1 | 0  5 | 27.82 | L-norleucine | **112 > 69**  69 > 41.1 | 0  5 | 16,74 |
| 28 | L-threonine | **161 > 129.1**  129 > 101.1 | 0  5 | 27.99 | D-β-leucine | **174 > 60**  74 > 43.1 | 0  5 | 16,97 |
| 29 | DL-aspartic acid | **113 > 85**  86 > 58 | 5  10 | 28.3 | L-β-leucine | **174 > 60**  74 > 43.1 | 5  10 | 17,19 |
| 30 | D-serine | **161 > 129.1**  132 > 60.1 | 0  10 | 29.53 | GABA | **100 > 44**  74 > 43 | 0  10 | 17,41 |
| 31 | L-serine | **161 > 129.1**  132 > 60.1 | 0  10 | 29.74 | L-aspartic acid | **113 > 85**  86 > 58 | 0  10 | 18,82 |
| 32 | D-homoserine | **128 > 100.1**  115 > 56 | 0  5 | 30.06 | D-aspartic acid | **113 > 85**  86 > 58 | 0  5 | 19,04 |
| 33 | L-homoserine | **128 > 100.1**  115 > 56 | 0  5 | 30.12 | L-threonine | **161 > 129.1**  129 > 101.1 | 0  5 | 24,58 |
| 34 | 5-aminovaleric acid | **130 > 98.1**  114 > 86.1 | 5  5 | 30.82 | D-threonine | **161 > 129.1**  129 > 101.1 | 5  5 | 24,7 |
| 35 | DL-glutamic acid | **188 > 128.1**  84 > 56 | 5  10 | 32.26 | D-methionine | **161 > 101.1**  129 > 101 | 5  10 | 26,24 |
| 36 | D-methionine | **161 > 101.1**  129 > 101 | 10  5 | 32.51 | L-methionine | **161 > 101.1**  129 > 101 | 5  10 | 26,59 |
| 37 | L-methionine | **161 > 101.1**  129 > 101 | 10  5 | 32.65 | D-phenylalanine | **91 > 65**  91 > 39.1 | 45  15 | 28,71 |
| 38 | DL-isoserine | **173 > 114**  86 > 58 | 5  5 | 35.2 | L-phenylalanine | **91 > 65**  91 > 39.1 | 45  15 | 28,93 |
| 39 | DL-phenylalanine | **91 > 65**  91 > 39.1 | 20  35 | 36.2 |  |  |  |  |

**Table S4**

BCF/MeOH derivatives analysed by GC-MS/MS (MRM transitions and retention times)

| N° | Compound | Chirasil-Dex | | Retention times (min) | Compound | Chirasil-L-Val | | Retention times (min) |
| --- | --- | --- | --- | --- | --- | --- | --- | --- |
|  |  | Transitions | CE (eV) |  |  | Transitions | CE (eV) |  |
| 1 | sarcosine | **144 > 57.1**  144 > 44 | 5  5 | 13.7 | sarcosine | **144 > 57.1**  144 > 44 | 5  5 | 12.06 |
| 2 | 2-AIBA | **158 > 58.1**  84 > 56 | 5  10 | 14.54 | DL-isovaline | **172 > 72.1**  98 > 55.1 | 10  10 | 12.06 |
| 3 | N-ethylglycine | **158 > 57.1**  158 > 41.1 | 5  20 | 14.74 | N-ethylglycine | **158 > 57.1**  158 > 41.1 | 5  20 | 13.72 |
| 4 | D-alanine | **144 > 44**  70 > 42.1 | 5  10 | 15.13 | 2-AiBA | **158 > 58.1**  84 > 56 | 5  10 | 14.9 |
| 5 | L-alanine | **144 > 44**  70 > 42.1 | 5  10 | 15.25 | D-alanine | **144 > 44**  70 > 42.1 | 5  10 | 15.31 |
| 6 | DL-isovaline | **172 > 72.1**  98 > 55.1 | 10  10 | 15.78 | L-alanine | **144 > 44**  70 > 42.1 | 5  10 | 15.74 |
| 7 | L-2ABA | **84 > 56**  84 > 41.1 | 10  5 | 16.82 | glycine | **130 > 57**  88 > 44.1 | 5  5 | 16.83 |
| 8 | D-2ABA | **84 > 56**  84 > 41.1 | 10  5 | 16.94 | GABA | **100 > 44**  74 > 43 | 25  5 | 16.87 |
| 9 | L-valine | **172 > 116**  98 > 55 | 5  5 | 17.33 | D-2ABA | **84 > 56**  84 > 41.1 | 10  5 | 17.76 |
| 10 | D-valine | **172 > 116**  98 > 55 | 5  5 | 17.58 | L-2ABA | **84 > 56**  84 > 41.1 | 10  5 | 18.18 |
| 11 | glycine | **130 > 57**  88 > 44.1 | 5  5 | 18.48 | D-valine | **172 > 116**  98 > 55 | 5  5 | 18.54 |
| 12 | DL-3ABA | **116 > 84.1**  116 > 42.1 | 5  20 | 18.83 | L-valine | **172 > 116**  98 > 55 | 5  5 | 18.89 |
| 13 | L-norvaline | **172 > 72**  98 > 55.1 | 5  10 | 19.24 | DL-3ABA* | **116 > 84.1**  116 > 42.1 | 5  20 | 19.66 |
| 14 | D-norvaline | **172 > 72**  98 > 55.1 | 5  10 | 19.35 | DL-3AIBA* | **112 > 56**  88 > 57 | 15  5 | 19.66 |
| 15 | L-alloisoleucine | **130 > 74**  115 > 83 | 5  5 | 19.63 | D-proline | **170 > 70.1**  114 > 70.1 | 10  10 | 20.78 |
| 16 | D-alloisoleucine | **130 > 74**  115 > 83 | 5  5 | 19.83 | L-proline | **170 > 70.1**  114 > 70.1 | 10  10 | 20.89 |
| 17 | D-3AIBA | **112 > 56**  88 > 57 | 15  5 | 20.12 | D-norvaline | **172 > 72**  98 > 55.1 | 5  10 | 20.95 |
| 18 | L-isoleucine | **130 > 74**  115 > 83 | 5  5 | 20.12 | D-alloisoleucine | **130 > 74**  115 > 83 | 5  5 | 21.28 |
| 19 | D-isoleucine | **130 > 74**  115 > 83 | 5  5 | 20.25 | L-norvaline | **172 > 72**  98 > 55.1 | 5  10 | 21.4 |
| 20 | L-leucine | **186 > 86**  112 > 69.1 | 5  5 | 20.39 | pentadecane | **71 > 43.1**  57 > 41.1 | 5  10 | 13.08 |
| 21 | L-3AIBA | **112 > 56**  88 > 57 | 15  5 | 20.55 | L-alloisoleucine | **130 > 74**  115 > 83 | 5  5 | 21.65 |
| 22 | D-leucine | **186 > 86**  112 > 69.1 | 5  5 | 20.64 | D-isoleucine | **130 > 74**  115 > 83 | 5  5 | 21.86 |
| 23 | D-proline | **170 > 70.1**  114 > 70.1 | 10  10 | 21.18 | L-isoleucine | **130 > 74**  115 > 83 | 5  5 | 22.16 |
| 24 | DL-β-leucine | **202 > 102.1**  74 > 43.1 | 5  5 | 21.41 | D-leucine | **186 > 86**  112 > 69.1 | 5  5 | 22.74 |
| 25 | L-proline | **170 > 70.1**  114 > 70.1 | 10  10 | 21.51 | L-leucine | **186 > 86**  112 > 69.1 | 5  5 | 23.22 |
| 26 | L-norleucine | **112 > 69**  69 > 41.1 | 5  5 | 22.09 | D-norleucine | **112 > 69**  69 > 41.1 | 5  5 | 23.98 |
| 27 | D-norleucine | **112 > 69**  69 > 41.1 | 5  5 | 22.2 | L-norleucine | **112 > 69**  69 > 41.1 | 5  5 | 24.38 |
| 28 | pentadecane | **71 > 43.1**  57 > 41.1 | 5  10 | 24.24 | D-β-leucine | **202 > 102.1**  74 > 43.1 | 5  5 | 24.31 |
| 29 | GABA | **100 > 44**  74 > 43 | 25  5 | 24.68 | L-β-leucine | **202 > 102.1**  74 > 43.1 | 5  5 | 24.45 |
| 30 | L-threonine | **133 > 101.1**  101 > 83 | 0  5 | 25.22 | L-aspartic acid | **202 > 102.1**  102 > 43.1 | 5  10 | 27.26 |
| 31 | D-threonine | **133 > 101.1**  101 > 83 | 0  5 | 25.36 | D-aspartic acid | **202 > 102.1**  102 > 43.1 | 5  10 | 27.44 |
| 32 | DL-aspartic acid | **202 > 102.1**  102 > 43.1 | 5  10 | 25.92 | D-homoserine | **146 > 102.1**  101 > 83.1 | 0  5 | 30.13 |
| 33 | L-serine | **101 > 83**  86 > 42 | 5  10 | 26.74 | L-homoserine | **146 > 102.1**  101 > 83.1 | 0  5 | 30.36 |
| 34 | D-serine | **101 > 83**  86 > 42 | 5  10 | 26.86 | DL-serine | **101 > 83**  86 > 42 | 5  10 | 31.03 |
| 35 | DL-homoserine | **146 > 102.1**  101 > 83.1 | 0  5 | 27.56 | 5-amino valeric acid | **130 > 98.1**  114 > 86.1 | 5  5 | 31.09 |
| 36 | 5-amino valeric acid | **130 > 98.1**  114 > 86.1 | 5  5 | 27.81 | L-threonine | **133 > 101.1**  101 > 83 | 0  5 | 31.25 |
| 37 | L-methionine | **189 > 101.1**  101 > 83 | 5  5 | 29.96 | D-threonine | **133 > 101.1**  101 > 83 | 0  5 | 31.38 |
| 38 | D-methionine | **189 > 101.1**  101 > 83 | 5  5 | 30.11 | DL-glutamic acid | **188 > 128.1**  84 > 56 | 5  10 | 32.99 |
| 39 | DL-glutamic acid | **188 > 128.1**  84 > 56 | 5  10 | 30.78 | D-methionine | **189 > 101.1**  101 > 83 | 5  5 | 32.4 |
| 40 | L-phenylalanine | **162 > 103.1**  91 > 65 | 25  15 | 34.5 | L-methionine | **189 > 101.1**  101 > 83 | 5  5 | 32.75 |
| 41 | D-phenylalanine | **162 > 103.1**  91 > 65 | 25  15 | 34.63 | D-phenylalanine | **162 > 103.1**  91 > 65 | 25  15 | 34.87 |
|  |  |  |  |  | L-phenylalanine | **162 > 103.1**  91 > 65 | 25  15 | 35.1 |

**Table S5**

IBCF/MeOH derivatives analysed by GC-MS/MS (MRM transitions and retention times)

| N° | Compound | Chirasil-Dex | | Retention times (min) | Compound | Chirasil-L-Val | | Retention times (min) |
| --- | --- | --- | --- | --- | --- | --- | --- | --- |
|  |  | Transitions | CE (eV) |  |  | Transitions | CE (eV) |  |
| 1 | sarcosine | **144 > 57.1**  144 > 44 | 5  5 | 21.12 | sarcosine | **144 > 57.1**  144 > 44 | 5  5 | 17.23 |
| 2 | 2-AIBA | **158 > 58.1**  84 > 56 | 5  10 | 21.59 | DL-isovaline | **172 > 72.1**  98 > 55.1 | 10  10 | 17.31 |
| 3 | N-ethylglycine | **158 > 57.1**  158 > 41.1 | 5  20 | 22.92 | N-ethylglycine | **158 > 57.1**  158 > 41.1 | 5  20 | 18.89 |
| 4 | D-alanine | **144 > 44**  70 > 42.1 | 5  10 | 23.32 | 2-AiBA | **158 > 58.1**  84 > 56 | 5  10 | 20,14 |
| 5 | L-alanine | **144 > 44**  70 > 42.1 | 5  10 | 23.54 | D-alanine | **144 > 44**  70 > 42.1 | 5  10 | 20,39 |
| 6 | L-isovaline | **172 > 72.1**  98 > 55.1 | 10  10 | 26.65 | L-alanine | **144 > 44**  70 > 42.1 | 5  10 | 20,86 |
| 7 | D-isovaline | **172 > 72.1**  98 > 55.1 | 10  10 | 26.81 | glycine | **130 > 57**  88 > 44.1 | 5  5 | 21,72 |
| 8 | L-2ABA | **84 > 56**  84 > 41.1 | 10  5 | 26.88 | GABA | **100 > 44**  74 > 43 | 25  5 | 21,84 |
| 9 | D-2ABA | **84 > 56**  84 > 41.1 | 10  5 | 27.22 | D-2ABA | **84 > 56**  84 > 41.1 | 10  5 | 22,69 |
| 10 | L-valine | **172 > 116**  98 > 55 | 5  5 | 28.4 | L-2ABA | **84 > 56**  84 > 41.1 | 10  5 | 23,11 |
| 11 | D-valine | **172 > 116**  98 > 55 | 5  5 | 28.7 | D-valine | **172 > 116**  98 > 55 | 5  5 | 23,47 |
| 12 | glycine | **130 > 57**  88 > 44.1 | 5  5 | 28.9 | L-valine | **172 > 116**  98 > 55 | 5  5 | 23,83 |
| 13 | L-norvaline | **172 > 72**  98 > 55.1 | 5  10 | 29.58 | DL-3ABA | **116 > 84.1**  116 > 42.1 | 5  20 | 24,51 |
| 14 | D-norvaline | **172 > 72**  98 > 55.1 | 5  10 | 29.83 | DL-3AIBA | **112 > 56**  88 > 57 | 15  5 | 24,58 |
| 15 | L-alloisoleucine | **186 > 130**  129 > 101 | 5  5 | 30.12 | D-proline | **170 > 70.1**  114 > 70.1 | 10  10 | 25,67 |
| 16 | D-alloisoleucine | **186 > 130**  129 > 101 | 5  5 | 30.4 | L-proline | **170 > 70.1**  114 > 70.1 | 10  10 | 25,79 |
| 17 | D-3AIBA | **112 > 56**  88 > 57 | 15  5 | 30.67 | D-norvaline | **172 > 72**  98 > 55.1 | 5  10 | 25.91 |
| 18 | L-isoleucine | **186 > 130**  129 > 101 | 5  5 | 30.83 | D-alloisoleucine | **130 > 74**  115 > 83 | 5  5 | 26,16 |
| 19 | D-isoleucine + L-leucine + L-3AiBA | **186 > 130**  129 > 101 | 5  5 | 31.1 | L-norvaline | **172 > 72**  98 > 55.1 | 5  10 | 26,51 |
| 20 | D-leucine | **186 > 130.2**  129 > 101.1 | 5  5 | 31.52 | pentadecane | **71 > 43.1**  57 > 41.1 | 5  10 | 13.08 |
| 21 | D-proline | **170 > 70.1**  114 > 70.1 | 10  10 | 32.04 | L-alloisoleucine | **130 > 74**  115 > 83 | 5  5 | 26.67 |
| 22 | DL-β-leucine | **142 > 96**  88.1 > 61 | 5  5 | 32.65 | D-isoleucine | **130 > 74**  115 > 83 | 5  5 | 26.88 |
| 23 | L-proline | **170 > 70.1**  114 > 70.1 | 10  10 | 32.53 | L-isoleucine | **130 > 74**  115 > 83 | 5  5 | 27.06 |
| 24 | L-norleucine | **186 > 86.2**  112 > 69 | 5  5 | 33.32 | D-leucine | **186 > 86**  112 > 69.1 | 5  5 | 27,63 |
| 25 | D-norleucine | **186 > 86.2**  112 > 69 | 5  5 | 33.51 | L-leucine | **186 > 86**  112 > 69.1 | 5  5 | 28.35 |
| 26 | pentadecane | **71 > 43.1**  57 > 41.1 | 5  10 | 24.24 | D-norleucine | **112 > 69**  69 > 41.1 | 5  5 | 28.91 |
| 27 | GABA | **130 > 84**  88 > 61 | 10  5 | 35.98 | L-norleucine | **112 > 69**  69 > 41.1 | 5  5 | 29.27 |
| 28 | L-threonine | **130 > 74**  101 > 83 | 5  5 | 38.34 | D-β-leucine | **202 > 102.1**  74 > 43.1 | 5  5 | 29.36 |
| 29 | D-threonine | **130 > 74**  101 > 83 | 5  5 | 38.52 | L-β-leucine | **202 > 102.1**  74 > 43.1 | 5  5 | 29.52 |
| 30 | DL-aspartic acid | **202 > 102.1**  102 > 43.1 | 5  10 | 39.46 | L-aspartic acid | **202 > 102.1**  102 > 43.1 | 5  10 | 32.64 |
| 31 | L-methionine | **203 > 101**  101 > 83 | 10  5 | 41.98 | D-aspartic acid | **202 > 102.1**  102 > 43.1 | 5  10 | 32.86 |
| 32 | D-methionine | **203 > 101**  101 > 83 | 10  5 | 42.14 | D-homoserine | **146 > 102.1**  101 > 83.1 | 0  5 | 35.22 |
| 33 | DL-glutamic acid | **188 > 128.1**  84 > 56 | 5  10 | 42.9 | L-homoserine | **146 > 102.1**  101 > 83.1 | 0  5 | 35.31 |
| 34 | L-phenylalanine | **176 > 131.1**  91 > 65 | 15  20 | 46.72 | DL-serine | **101 > 83**  86 > 42 | 5  10 | 35.94 |
| 35 | D-phenylalanine | **176 > 131.1**  91 > 65 | 15  20 | 49.56 | 5-amino valeric acid | **130 > 98.1**  114 > 86.1 | 5  5 | 36.06 |
| 36 |  |  |  |  | L-threonine | **133 > 101.1**  101 > 83 | 0  5 | 36.32 |
| 37 |  |  |  |  | D-threonine | **133 > 101.1**  101 > 83 | 0  5 | 36.45 |
| 38 |  |  |  |  | DL-glutamic acid | **188 > 128.1**  84 > 56 | 5  10 | 38.04 |
| 39 |  |  |  |  | D-methionine | **189 > 101.1**  101 > 83 | 5  5 | 37.59 |
| 40 |  |  |  |  | L-methionine | **189 > 101.1**  101 > 83 | 5  5 | 37.86 |
| 41 |  |  |  |  | D-phenylalanine | **162 > 103.1**  91 > 65 | 25  15 | 38.99 |
| 42 |  |  |  |  | L-phenylalanine | **162 > 103.1**  91 > 65 | 25  15 | 39.18 |

**Table S6**

Limits of detection of amino acid derivatives by GC-MS/MS analysis (mol/L)

|  | Chirasil-Dex | | | | Chirasil-L-Val | | | | |
| --- | --- | --- | --- | --- | --- | --- | --- | --- | --- |
|  | **MCF/MeOH** | **ECF/MeOH** | **BCF/MeOH** | **IBCF/MeOH** | **MCF/MeOH** | **ECF/MeOH** | **BCF/MeOH** | **IBCF/MeOH** | **ECF/HFB** |
| sarcosine | 1,89.10^-07^ | 8,21.10^-07^ | 3,71.10^-07^ | 3,46.10^-07^ | 6,11.10^-06^ | 9,24.10^-07^ | 8,05.10^-07^ | 9,49.10^-07^ | 1,36.10^-07^ |
| 2AIBA | 9,57.10^-07^ | 1,15.10^-06^ | 1,54.10^-07^ | 1,92.10^-07^ | 5,7.10^-06^ | 3,90.10^-07^ | 1,76.10^-06^ | 2,27.10^-06^ | 2,46.10^-07^ |
| alanine | 5,12.10^-07^ | 2,36.10^-06^ | 1,12.10^-07^ | 1,94.10^-07^ | 3,11.10^-06^ | 8,55.10^-07^ | 2,38.10^-07^ | 2,96.10^-07^ | 1,82.10^-06^ |
| N-ethylglycine | 1,03.10^-07^ | 1,39.10^-05^ | 3,61.10^-07^ | 3,88.10^-07^ | 3,49.10^-07^ | 7,9.10^-06^ | 4,04.10^-07^ | 2,55.10^-07^ | 1,59.10^-07^ |
| isovaline | 4,40.10^-07^ | 1,60.10^-06^ | 7,57.10^-07^ | 8,34.10^-07^ | 2,63.10^-07^ | 5,83.10^-07^ | 5,4.10^-07^ | 5,3.10^-07^ | 6,08.10^-07^ |
| 2ABA | 8,63.10^-07^ | 1,46.10^-06^ | 6,25.10^-07^ | 6,73.10^-07^ | 2,07.10^-07^ | 9,03.10^-07^ | 2,66.10^-07^ | 3,48.10^-07^ | 7,35.10^-08^ |
| valine | 2,35.10^-07^ | 1,65.10^-06^ | 5,28.10^-07^ | 6,16.10^-07^ | 7,22.10^-08^ | 9,87.10^-07^ | 1,21.10^-07^ | 3,16.10^-07^ | 4,13.10^-07^ |
| glycine | 4,80.10^-06^ | 6,10.10^-06^ | 2,09.10^-07^ | 2,49.10^-07^ | 3,47.10^-06^ | 7,59.10^-06^ | 8,44.10^-07^ | 8.10^-06^ | 3,9.10^-06^ |
| 3ABA | 1,66.10^-06^ | nd | 2,05.10^-06^ | 2,73.10^-06^ | 5,79.10^-07^ | nd | 3,54.10^-07^ | 2,52.10^-07^ | nd |
| norvaline | 3,10.10^-07^ | 1,91.10^-06^ | 4,63.10^-07^ | 4,33.10^-07^ | 8,45.10^-08^ | nd | 7,17.10^-08^ | 9,04.10^-08^ | 2,46.10^-07^ |
| alloisoleucine | 6,70.10^-07^ | 8,06.10^-07^ | 4,94.10^-07^ | 4,78.10^-07^ | 7,89.10^-08^ | 8,22.10^-08^ | 8,65.10^-08^ | 7,96.10^-08^ | 3,66.10^-07^ |
| isoleucine | 3,58.10^-07^ | 7,14.10^-07^ | 6,99.10^-07^ | 5,42.10^-07^ | 6,54.10^-08^ | 5,19.10^-08^ | 9,47.10^-08^ | 9,33.10^-08^ | 3,17.10^-07^ |
| leucine | 3,10.10^-07^ | 9,23.10^-07^ | 3,74.10^-07^ | 4.57.10^-07^ | 5,91.10^-08^ | 7,35.10^-08^ | 8,19.10^-08^ | 7,15.10^-08^ | 3,48.10^-07^ |
| β-alanine | 2,90.10^-06^ | 3,55.10^-06^ | nd | nd | 7,55.10^-07^ | nd | 5.98.10^-6^ | 8.41.10^-6^ | nd |
| 3AIBA | 2,03.10^-06^ | 2,31.10^-06^ | 2,60.10^-06^ | 2,68.10^-06^ | 4,85.10^-07^ | 6,96.10^-07^ | 3,1.10^-07^ | 4,07.10^-07^ | 4,7.10^-06^ |
| β-leucine | 7,66.10^-07^ | 4,37.10^-06^ | 8,88.10^-07^ | 8,1.10^-07^ | 1,68.10^-07^ | 2,13.10^-06^ | 4,59.10^-06^ | 2,5.10^-06^ | nd |
| norleucine | 3,97.10^-07^ | 6,33.10^-07^ | 1,48.10^-07^ | 3,93.10^-07^ | 6,97.10^-08^ | 3,1.10^-08^ | 2,81.10^-08^ | 3,66.10^-08^ | 4,19.10^-07^ |
| proline | 5,47.10^-07^ | 5,91.10^-07^ | 5,62.10^-07^ | 5,8.10^-07^ | 8,15.10^-07^ | 1,67.10^-06^ | 1,95.10^-06^ | 2,41.10^-06^ | 2,32.10^-07^ |
| GABA | 1,92.10^-05^ | 7,18.10^-06^ | 6,41.10^-06^ | 6,37.10^-06^ | 1,17.10^-05^ | 2,49.10^-05^ | 4,2.10^-05^ | 6,79.10^-05^ | 4,58.10^-06^ |
| threonine | 8,70.10^-05^ | 3,17.10^-05^ | 7,02.10^-05^ | 7,73.10^-05^ | 5,93.10^-06^ | nd | 4,69.10^-06^ | 3,69.10^-06^ | nd |
| aspartic acid | 4,11.10^-06^ | 2,93.10^-06^ | 6,97.10^-07^ | 6,89.10^-07^ | 2,62.10^-06^ | 2,88.10^-06^ | 5,15.10^-06^ | 3,71.10^-06^ | 9,08.10^-06^ |
| homoserine | 3,81.10^-05^ | 1,60.10^-05^ | 5,38.10^-06^ | 4,98.10^-06^ | 5,03.10^-05^ | 6,4.10^-05^ | 9,07.10^-05^ | 6,34.10^-05^ | nd |
| 5-amino valeric acid | 1,94.10^-05^ | 6,13.10^-06^ | 1,72.10^-05^ | 1,61.10^-05^ | 2,81.10^-05^ | nd | 2,11.10^-05^ | 3,63.10^-05^ | 2,04.10^-05^ |
| glutamic acid | 3,87.10^-05^ | 5,52.10^-06^ | 4,1.10^-06^ | 4,86.10^-06^ | 1,7.10^-05^ | 3,25.10^-05^ | 2,38.10^-05^ | 1,06.10^-05^ | 5,31.10^-05^ |
| methionine | 2,06.10^-06^ | 1,74.10^-06^ | 3,17.10^-06^ | 3,45.10^-06^ | 8,41.10^-07^ | 6,44.10^-07^ | 8,21.10^-07^ | 7,42.10^-07^ | 6,96.10^-07^ |
| phenylalanine | 9,10.10^-08^ | 6,44.10^-07^ | 2,84.10^-07^ | 2,75.10^-07^ | 2,17.10^-08^ | 4,91.10^-07^ | 2,35.10^-08^ | 4,12.10^-08^ | 5,15.10^-08^ |

nd : not detected

References

[1] C. Serra, J. Lange, Q.B. Remaury, R. Timoumi, G. Danger, B. Laurent, L. Remusat, C.G. Rodier, P. Poinot, Integrative analytical workflow to enhance comprehensive analysis of organic molecules in extraterrestrial objects, Talanta (2022) 123324. https://doi.org/10.1016/j.talanta.2022.123324.
